# Effect of manual handling weight for lifting and carrying on the severity of acute occupational low back pain

**DOI:** 10.1007/s00420-025-02148-5

**Published:** 2025-05-24

**Authors:** Kazuyuki Iwakiri, Keiichi Miki, Takeshi Sasaki

**Affiliations:** National Institute of Occupational Safety and Health, Nagao 6-21-1, Tama-Ku, Kawasaki, 214-8585 Japan

**Keywords:** Absenteeism, Low back pain, Manual labor, Severity of illness index

## Abstract

**Purpose:**

Preventing the progression of occupational low back pain (LBP) is a critical occupational safety and health concern, alongside reducing its incidence. Manual handling of heavy loads may increase LBP severity. This study investigates the impact of lifting and carrying weights on LBP severity in affected workers.

**Methods:**

A total of 2418 cases of acute occupational LBP, each resulting in more than four days of absence from work, were analyzed. These cases, reported as industrial accidents in Japan 2018–2019, were categorized into four weight-handling groups: < 10, 10–20, 20–30, and ≥ 30 kg. LBP severity was defined based on the duration of work absence, as determined by a physician’s diagnosis at the onset, and was categorized into four groups: 4–7, 8–14, 15–30, and ≥ 31 days. Multinomial logistic regression analysis was conducted to assess the relationship between handling weights and absence duration.

**Results:**

The odds ratio (OR) for absence of ≥ 31 days compared with 4–7 days increased with heavier handling weights. Notably, workers handling 30 kg or more weights had a significantly higher OR than those under 10 kg (OR: 1.75; 95% CI: 1.11–2.77). The ORs for absence of 8–14 and 15–30 days compared with 4–7 days showed no significant association with handling weight.

**Conclusion:**

Lifting and carrying heavier loads were associated with increased LBP severity and prolonged work absences. Minimizing manual handling loads may be a practical strategy to reduce the severity of acute occupational LBP and prevent prolonged work absences.

**Supplementary Information:**

The online version contains supplementary material available at 10.1007/s00420-025-02148-5.

## Introduction

Preventing the progression of occupational low back pain (LBP) is a significant occupational safety and health challenge, equally as critical as preventing its occurrence (Khadour et al. 2024; Saravanan et al. 2021; Shigetoh et al. 2024; Wu et al. 2024). Addressing this issue facilitates the early return of employees to the workplace, thereby reducing workers’ compensation costs and maintaining labor productivity.

In Japan, occupational LBP accounts for approximately 60% of all occupational diseases (Ministry of Health, Labour and Welfare 2004–2023), with 16% of cases involving severe LBP that results in more than one month of absence from work (Iwakiri et al. 2022). Additionally, nearly a quarter of occupational LBP cases are attributed to manual handling of heavy objects, such as lifting and carrying (National Institute of Occupational Safety and Health, Japan 2021). As a preventive measure, the maximum allowable manual handling load is regulated at 40% of body weight for men and 24% for women, according to guidelines issued by the Ministry of Health, Labour and Welfare (2013). However, these national standards do not fully comply with ISO 11228–1 (International Organization for Standardization 2021).

The relationship between handling loads and LBP remains unclear (Bakker et al. 2009; Kwon et al. 2011; Wai et al. 2010). However, manual handling of heavy objects is recognized as a risk factor for LBP (Bláfoss et al. 2020, 2024; Brauer et al. 2020; Holtermann et al. 2013). Increased strain on lumbar intervertebral discs has been reported in association with heavier manual handling loads (Mondal et al. 2019; Schäfer et al. 2023; Schmid et al. 2020; Wilke et al. 1999). Consequently, heavier manual handling loads may increase the risk of severe occupational LBP. However, recent occupational health studies have not specifically investigated the relationship between manual handling weight and the severity of occupational LBP.

In Japan, worker accidents must be reported to the Ministry of Health, Labour and Welfare through the Reports of Worker Casualties. These reports categorize incidents into two groups: absences of four or more days and absences of fewer than four days. The former is considered more severe and requires details such as the number of absent days, the circumstances surrounding the incident, and its causes. The reported absence duration is based on the recuperation period determined by a physician’s diagnosis, with longer absences indicating greater injury severity. Reports on manual lifting and carrying accidents include descriptions of work conditions, such as handling weight and working posture, documented in free-form text and diagrams.

Therefore, this study aimed to clarify the impact of manual handling weight during lifting and carrying on the severity of occupational LBP, as indicated by the number of absent days from work.

## Methods

### Research design

This study analyzed 10,208 cases of compensable occupational LBP documented in the Reports of Worker Casualties. These cases involved four or more days of absence and were reported as industrial accidents by the Ministry of Health, Labour and Welfare in Japan between 2018 and 2019. The cases analyzed represented 60.1% (*N* = 10,208) of all 16,994 occupational disease cases. Of these, 99.4% (*N* = 10,148) were classified as acute (accident-related) LBP and 0.6% (*N* = 60) as chronic (non-accident-related) LBP (Iwakiri et al. 2022). This study focused on acute LBP, which constituted the majority of cases and was primarily caused by back strain while lifting and lowering heavy loads.

**Fig. 1:**
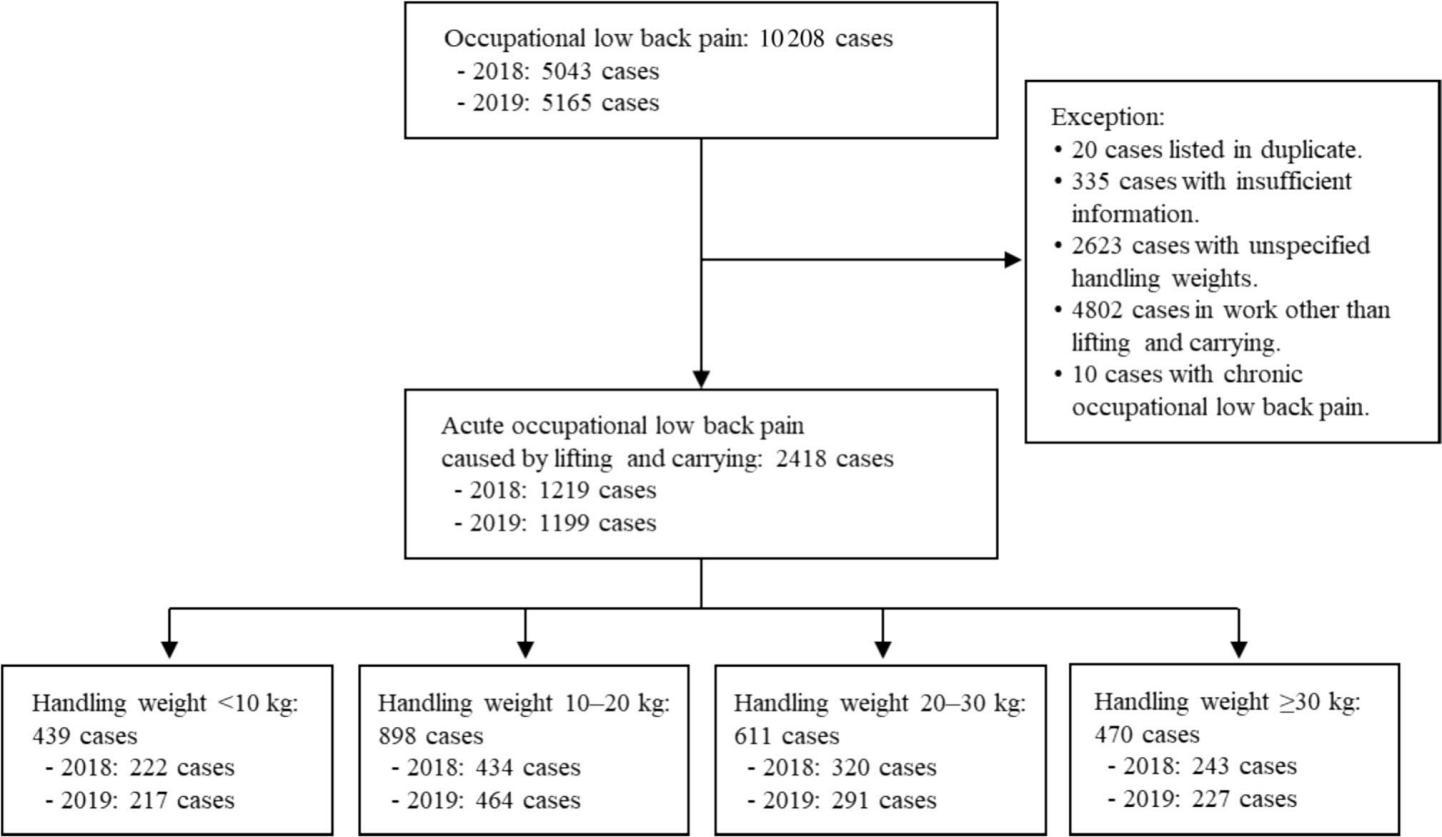
Flowchart illustrating the selection process in this study

The dataset was refined by excluding 20 duplicate cases, 335 cases with insufficient information, 2623 cases with unspecified handling weights, 4802 cases involving activities other than lifting and carrying, and 10 cases of chronic occupational LBP (Fig. 1). As a result, the final dataset comprised 2418 cases, which were categorized into four handling weight groups.

This study was performed in line with the principles of the Declaration of Helsinki and adopted an opt-out approach, enabling individuals to decline participation and publication without providing a reason. Ethical approval was obtained from the Ethics Board of the National Institute of Occupational Safety and Health (Registration ID: 2021N07). The study was supported by funding from the institute (Grant N-P03-01).

### Reports of worker casualties

The Reports of Worker Casualties for cases involving ≥ 4 days of absence included victim details, such as gender, age, years of experience, duration of work absence, industry type, and circumstances and causes of the accident. Years of experience were defined as the total years of employment in the worker’s current occupation. Work absence was defined as the expected recovery period determined by a physician’s diagnosis (Katoh et al. 2019). The duration of work absence, as determined by physician diagnosis, was used to define LBP severity and was classified into four categories: 4–7, 8–14, 15–30, and ≥ 31 days. The circumstances and causes of accidents were recorded in free-form text and diagrams, from which information on handling weights and working postures was extracted.

Lifting and carrying operations were defined as tasks involving lifting, lowering, and carrying objects weighing > 0 kg, while activities such as pushing, pulling, and rolling objects were excluded. For tasks performed by multiple workers, the total weight was divided by the number of workers to estimate the weight handled per individual. Handling weights were categorized into four groups: < 10 kg (*N* = 439), 10–20 kg (*N* = 898), 20–30 kg (*N* = 611), and ≥ 30 kg (*N* = 470; Fig. 1). Working postures were categorized as either proper posture (without improper movements) or improper postures, which included twisting, bending forward, squatting, and other unnatural positions.

### Data analysis

The chi-squared (*χ*^2^) test was used to compare four weight groups: < 10, 10–20, 20–30, and ≥ 30 kg. Multinomial logistic regression analysis was performed to examine the association between handling weights and the number of absent days from work. Odds ratios (ORs) with 95% confidence intervals (95% CIs) were calculated for the model.

The dependent variable was the number of absent days from work, categorized into three groups: 8–14, 15–30, and ≥ 31 days, with 4–7 days as the reference category. The independent variable was handling weight, categorized into 10–20, 20–30, and ≥ 30 kg, with < 10 kg as the reference category. The model was adjusted for sex, age, industry type, and working posture, all treated as categorical variables.

The variance inflation factor for the independent and adjusted variables was < 1.2, confirming the absence of multicollinearity. All statistical analyses were conducted using IBM SPSS Statistics version 27, with a statistical significance set at *p* < 0.05 for all tests.

## Results

The mean age of the injured workers was 40.8 yr. (standard deviation [*SD*]: 12.6 yr.; range: 16–90 yr.). Their mean years of experience was 5.2 yr. (*SD*: 7.1 yr.; range: 0–50 yr.), and their mean number of absent days from work was 22.5 days (*SD*: 28.3 days; range: 4–364 days).

Working handling heavier weights were more likely to be male, work in proper postures, and be employed in the construction and transportation industries compared with those handling lighter weights (Table 1). Regarding absent days from work, those handling heavier weights were less likely to have absences of 4–7 days and more likely to have absences of ≥ 31 days. No significant differences in age or years of experience were observed among the weight groups.

**Table 1:** Number of occupational low back pain cases by worker basic information and handling weight category (*N* = 10,208)

*N* (%)	Handling weight				*p* value
	< 10 kg (*N* = 439)	10–20 kg (*N* = 898)	20–30 kg (*N* = 611)	≥ 30 kg (*N* = 470)	
Sex					< 0.001
Male	220 (50.1)	514 (57.2)	470 (76.9)	427 (90.9)	
Female	219 (49.9)	384 (42.8)	141 (23.1)	43 (9.1)	
Age (years)					0.802
< 30	95 (21.6)	184 (20.5)	132 (21.6)	97 (20.6)	
30–39	107 (24.4)	238 (26.5)	161 (26.4)	144 (30.6)	
40–49	127 (28.9)	257 (28.6)	166 (27.2)	118 (25.1)	
50–59	66 (15.0)	140 (15.6)	94 (15.4)	76 (16.2)	
≥ 60	44 (10.0)	79 (8.8)	58 (9.5)	35 (7.4)	
Years of experience					0.429
< 1	135 (30.8)	272 (30.3)	174 (28.5)	138 (29.7)	
1–3	110 (25.1)	207 (23.1)	139 (22.7)	85 (18.1)	
3–5	61 (13.9)	113 (12.6)	79 (12.9)	70 (14.9)	
5–10	66 (15.0)	144 (16.0)	98 (16.0)	84 (17.9)	
≥ 10	67 (15.3)	162 (18.0)	121 (19.8)	93 (19.8)	
Industry					< 0.001
Manufacturing	103 (23.5)	252 (28.1)	188 (30.8)	119 (25.3)	
Construction	12 (2.7)	27 (3.0)	41 (6.7)	48 (10.2)	
Transportation	43 (9.8)	124 (13.8)	100 (16.4)	104 (22.1)	
Forwarder	13 (3.0)	36 (4.0)	18 (2.9)	12 (2.6)	
Commerce	173 (39.4)	271 (30.2)	140 (22.9)	74 (15.7)	
Hospitality and entertainment industry	39 (8.9)	62 (6.9)	31 (5.1)	28 (6.0)	
Cleaning and slaughter industry	16 (3.6)	20 (2.2)	19 (3.1)	20 (4.3)	
Other businesses	40 (9.1)	106 (11.8)	74 (12.1)	65 (13.8)	
Working posture					< 0.001
Proper posture	240 (54.7)	578 (64.4)	408 (66.8)	334 (71.1)	
Twisting posture	63 (14.4)	96 (10.7)	55 (9.0)	37 (7.9)	
Forward-bending position	34 (7.7)	77 (8.6)	36 (5.9)	20 (4.3)	
A half-crouching position	41 (9.3)	52 (5.8)	41 (6.7)	30 (6.4)	
Other postures	61 (13.9)	95 (10.6)	71 (11.6)	49 (10.4)	
Absent days from work					0.034
4–7	160 (36.4)	303 (33.7)	200 (32.7)	144 (30.6)	
8–14	128 (29.2)	274 (30.5)	155 (25.4)	125 (26.6)	
15–30	105 (23.9)	211 (23.5)	165 (27.0)	120 (25.5)	
≥ 31	46 (10.5)	110 (12.2)	91 (14.9)	81 (17.2)	

Multinomial logistic regression analysis indicated that the OR for absence of ≥ 31 days increased with heavier handling weights compared with 4–7 days, while no significant associations were found for absences of 8–14 or 15–30 days (Table 2). For absences of 31 days or more, workers handling weights of 30 kg or more had a significantly higher OR than those handling less than 10 kg (OR: 1.75; 95% CI: 1.11–2.77). Additionally, workers handling weights of 20–30 kg exhibited a tendency toward a higher OR than those handling less than 10 kg (OR: 1.51; 95% CI: 0.98–2.31). However, no significant difference in OR was observed between handling weights of 10–20 kg and less than 10 kg (OR: 1.28; 95% CI: 0.86–1.91).

**Table 2:** Association of absent days from work with handling weights using multinomial logistic regression analysis

	Absent days for 8–14 days (ref 4–7 days)	Absent days for 15–30 days (ref 4–7 days)	Absent days for ≥ 31 days (ref 4–7 days)
	OR (95% CI)	OR (95% CI)	OR (95% CI)
Handling weight (kg)			
< 10	1.00 (Reference)	1.00 (Reference)	1.00 (Reference)
10–20	1.11 (0.83–1.48)	1.05 (0.77–1.42)	1.28 (0.86–1.91)
20–30	0.92 (0.67–1.27)	1.16 (0.83–1.61)	1.51 (0.98–2.31)
≥ 30	1.00 (0.71–1.43)	1.10 (0.76–1.59)	1.75 (1.11–2.77)
(Adjusted variables)			
Sex			
Male	1.00 (Reference)	1.00 (Reference)	1.00 (Reference)
Female	0.95 (0.75–1.20)	0.94 (0.73–1.21)	0.87 (0.64–1.20)
Age (years)			
< 30	1.00 (Reference)	1.00 (Reference)	1.00 (Reference)
30–39	0.98 (0.73–1.31)	0.94 (0.69–1.28)	1.52 (1.01–2.28)
40–49	1.10 (0.82–1.48)	1.23 (0.91–1.67)	1.51 (1.00–2.28)
50–59	1.36 (0.97–1.92)	1.49 (1.04–2.14)	2.10 (1.33–3.32)
≥ 60	1.29 (0.82–2.03)	2.03 (1.31–3.17)	4.43 (2.66–7.37)
Industry			
Manufacturing	1.00 (Reference)	1.00 (Reference)	1.00 (Reference)
Construction	1.14 (0.65–2.02)	2.33 (1.40–3.88)	2.24 (1.21–4.15)
Transportation	1.08 (0.78–1.50)	1.08 (0.77–1.52)	1.50 (0.99–2.27)
Forwarder	1.25 (0.71–2.19)	1.05 (0.57–1.93)	0.34 (0.10–1.14)
Commerce	0.94 (0.71–1.25)	0.82 (0.61–1.10)	1.15 (0.79–1.66)
Hospitality and entertainment industry	1.13 (0.71–1.80)	1.42 (0.89–2.26)	2.04 (1.18–3.54)
Cleaning and slaughter industry	0.94 (0.49–1.80)	1.19 (0.64–2.24)	1.21 (0.56–2.61)
Other businesses	1.51 (1.06–2.16)	1.18 (0.81–1.73)	1.52 (0.95–2.41)
Working posture			
Proper posture	1.00 (Reference)	1.00 (Reference)	1.00 (Reference)
Twisting posture	0.95 (0.68–1.34)	0.76 (0.53–1.10)	0.95 (0.61–1.46)
Forward-bending position	0.69 (0.46–1.04)	0.73 (0.48–1.12)	0.65 (0.37–1.14)
A half-crouching position	0.93 (0.61–1.41)	1.05 (0.69–1.59)	0.78 (0.45–1.38)
Other postures	1.05 (0.75–1.46)	1.05 (0.74–1.48)	1.23 (0.82–1.86)

95% CI: and present the OR first, followed by the 95% CI. 95% confidence interval; *OR*: odds ratio. Adjusted variables are sex, age, industry, and working posture

## Discussion

This study examined the impact of handling weight during lifting and carrying on the severity of acute occupational LBP, as indicated by the duration of work absence. The findings demonstrated that handling weight was not associated with absences of ≤ 30 days but was associated with prolonged absences of ≥ 31 days, with the most pronounced impact observed for weights of ≥ 30 kg.

Although the precise mechanisms underlying acute LBP remain unclear, primary sources of pain are believed to include damage or inflammation of the intervertebral disk (Hyodo et al. 2005; Ohtori et al. 2015), facet joint (O’Neill et al. 2009), sacroiliac joint (Cohen 2005; Kaye et al. 2021), and muscle fascia (Brandl et al. 2022; Cozacov et al. 2022). Biomechanical research has demonstrated that increased manual load leads to higher compressive forces on the lumbar spine, which may aggravate damage to intervertebral discs, facet joints, sacroiliac joints, and associated muscle fascia (Mondal et al. 2019; Schäfer et al. 2023; Schmid et al. 2020; Wilke et al. 1999). Therefore, increased handling weight imposes greater stress on these structures, leading to more significant damage, which likely contributes to increased LBP severity and prolonged work absences.

This study found no significant relationship between handling weight and absence durations of 30 days or less. No standardized guidelines exist for determining the appropriate length of work absence, with clinicians typically making this decision in consultation with patients. Acute LBP, commonly referred to as a strained back, generally resolves within a few days to 4 weeks (Maher et al. 2017; Pengel et al. 2003), though in some cases, it may persist beyond a month (Itz et al. 2012; McIntosh and Hall 2011; Pfeiffer et al. 2024). The factors contributing to prolonged LBP remain unclear, but possible causes include herniated disks (Kotwicki et al. 2021), spinal canal stenosis (Lai et al. 2021), and osteoporosis (Casazza 2012), as well as psychological and psychosocial influences (Hallegraeff et al. 2012; Nieminen et al. 2021). While further research is needed to clarify these aspects, cases of LBP that resolve within one month may not be significantly affected by handling weight.

Among the variables adjusted in the multinomial logistic regression analysis, age, and industry type were significantly associated with prolonged work absences (≥ 31 days, reference group: 4–7 days). The OR for longer absences increased with age, with the highest risk observed among workers aged ≥ 60 yr, despite their typically lighter manual handling loads. Prior studies suggest that age-related degeneration of intervertebral discs and spinal musculature contributes to this vulnerability (Asai et al. 2020; Benoist 2003; Dallaway et al. 2020a, 2020b; Ferguson and Steffen 2003). Regarding industry type, elevated ORs were noted in the construction and hospitality and entertainment sectors compared with the manufacturing sector. Automation in manufacturing has reduced manual load handling, whereas construction work still involves frequent handling of heavy materials such as cement bags and steel plates. Similarly, in the hospitality and entertainment industry, workers often lift items like food and alcoholic beverages in confined spaces, and staff shortages make adequate rest difficult. Prolonged standing and repetitive lifting likely contribute to musculoskeletal strain, particularly among older workers. These findings suggest that older workers in these sectors who are involved in heavy lifting face an increased risk of developing severe LBP.

This study has several limitations. First, not all occupational LBP cases are reported as industrial accidents. The process of filing claims for occupational injuries is often time-consuming, leading some individuals to forgo reporting. Consequently, this study only reflects cases where claims were submitted. Second, missing weight data in some reports may have influenced the findings, as claims were accepted even when weight values were not provided. The absence of this data may indicate different trends, and the results of this study apply only to cases with reported weight values. Third, this study focused exclusively on acute LBP and did not consider chronic LBP, which requires separate analysis. Lastly, the recorded absence period may not always correspond to the actual duration of absence. A previous study reported that approximately 70% of workers experienced longer absences than officially recorded (Katoh et al. 2019). As a result, this study may have underestimated LBP severity. Further research is needed to address these limitations and enhance the comprehensiveness of the findings.

Although this study has certain limitations, it also has notable strengths. It covers occupational LBP cases eligible for compensation nationwide in Japan. Additionally, because workers with load-handling duties and short absences served as the reference group, identifying statistically significant differences was more challenging. Nevertheless, the significant association between heavier handling weights and prolonged absences of ≥ 31 days is a noteworthy finding.

## Conclusion

Heavier lifting and carrying loads were associated with prolonged absences of ≥ 31 days but showed no significant relationship with absences of ≤ 30 days. This association was particularly pronounced for weights of ≥ 30 kg. Handling heavier manual loads is likely to exacerbate the severity of acute occupational LBP and result in extended work absences. Reducing load weights may help prevent the progression of acute LBP into more severe stages.

## Supplementary Information

Below is the link to the electronic supplementary material.

Supplementary file1 (DOCX 41 KB)

## Data Availability

Data will be available upon request.
