# Supplementary material for: Effect of manual handling weight for lifting and carrying on the severity of acute occupational low back pain
Source: Int Arch Occup Environ Health. 2025 May 24;98(6):507–13. doi: 10.1007/s00420-025-02148-5 (PMC12331803; doi:10.1007/s00420-025-02148-5)
Supplement: Supplementary file 1 — Supplementary file1 (DOCX 41 KB) [file 420_2025_2148_MOESM1_ESM.docx]

**Supporting Information Table S1.**

|  | Absent days for ≥31 days (ref 4–7 days)  OR (95% CI) | | | |
| --- | --- | --- | --- | --- |
|  | Crude | Model 1 | Model 2 | Model 3 |
| Handling weight (kg) |  |  |  |  |
| <10 | 1.00 (Reference) | 1.00 (Reference) | 1.00 (Reference) | 1.00 (Reference) |
| 10–20 | 1.26 (0.85–1.87) | 1.27 (0.85–1.89) | 1.28 (0.86–1.91) | 1.28 (0.86–1.91) |
| 20–30 | 1.58 (1.05–2.39) | 1.55 (1.01–2.36) | 1.52 (0.99–2.33) | 1.51 (0.98–2.31) |
| ≥30 | 1.96 (1.28–3.00) | 1.92 (1.23–3.00) | 1.78 (1.14–2.81) | 1.75 (1.11–2.77) |
| (*Adjusted variables*) |  |  |  |  |
| Sex |  |  |  |  |
| Male |  | 1.00 (Reference) | 1.00 (Reference) | 1.00 (Reference) |
| Female |  | 0.86 (0.64–1.17) | 0.88 (0.65–1.21) | 0.87 (0.64–1.20) |
| Age (yr) |  |  |  |  |
| <30 |  | 1.00 (Reference) | 1.00 (Reference) | 1.00 (Reference) |
| 30–39 |  | 1.47 (0.98–2.19) | 1.52 (1.01–2.27) | 1.52 (1.01–2.28) |
| 40–49 |  | 1.51 (1.00–2.26) | 1.52 (1.01–2.29) | 1.51 (1.00–2.28) |
| 50–59 |  | 2.02 (1.28–3.18) | 2.10 (1.33–3.31) | 2.10 (1.33–3.32) |
| ≥60 |  | 4.54 (2.75–7.50) | 4.43 (2.67–7.36) | 4.43 (2.66–7.37) |
| Industry |  |  |  |  |
| Manufacturing |  |  | 1.00 (Reference) | 1.00 (Reference) |
| Construction |  |  | 2.24 (1.21–4.13) | 2.24 (1.21–4.15) |
| Transportation |  |  | 1.51 (0.99–2.28) | 1.50 (0.99–2.27) |
| Forwarder |  |  | 0.34 (0.10–1.16) | 0.34 (0.10–1.14) |
| Commerce |  |  | 1.13 (0.78–1.64) | 1.15 (0.79–1.66) |
| Hospitality and  entertainment industry |  |  | 2.06 (1.19–3.56) | 2.04 (1.18–3.54) |
| Cleaning and slaughter  industry |  |  | 1.23 (0.57–2.65) | 1.21 (0.56–2.61) |
| Other businesses |  |  | 1.53 (0.96–2.43) | 1.52 (0.95–2.41) |
| Working posture |  |  |  |  |
| Proper posture |  |  |  | 1.00 (Reference) |
| Twisting posture |  |  |  | 0.95 (0.61–1.46) |
| Forward-bending  position |  |  |  | 0.65 (0.37–1.14) |
| A half-crouching  position |  |  |  | 0.78 (0.45–1.38) |
| Other postures |  |  |  | 1.23 (0.82–1.86) |

*Note*. 95% CI, 95% confidence interval; OR, odds ratio. Adjusted variables are sex and age in Model 1, sex, age, and industry in Model 2, and sex, age, industry, and working posture in Model 3.
